# Acute Effect of Single-Session Cerebellar Anodal Transcranial Direct Current Stimulation on Static and Dynamic Balance in Healthy Volunteers

**DOI:** 10.3390/brainsci13071107

**Published:** 2023-07-21

**Authors:** Ezgi Tuna Erdoğan, Can Kır, Esin Beycan, Esin Karakaya, Sanem Altınçınar, Türkü Bayramoğlu, Gökçer Eskikurt, Sacit Karamürsel

**Affiliations:** 1 Department of Physiology, Koç University School of Medicine, 34450 Istanbul, Turkey; 2 Innovative Center for Applied Neurosciences, Faculty of Medicine, Istinye University, 34010 Istanbul, Turkey

**Keywords:** cerebellar transcranial direct current stimulation, dynamic balance, static balance, healthy volunteers

## Abstract

Several studies have shown the positive effect of cerebellar transcranial direct current stimulation (ctDCS) on balance in patients and older adults. However, in healthy volunteers, the results are conflicting. We aimed to investigate the immediate effect of anodal ctDCS on the dynamic–static balance in healthy, non-athletic young adults due to the possible benefits for sports performance. Twenty-one healthy volunteers participated in two consecutive 20 min sessions of ctDCS (2 mA current intensity), with 1-week intervals (anodal ctDCS–sham ctDCS). Flamingo and Y-Balance tests were used to evaluate the static and dynamic balances before and after the ctDCS. A Continuous Performance Test (CPT) was used to evaluate the changes in sustained attention, impulsivity, and vigilance. A repeated measure analysis of variance (ANOVA) was used to compare the changes in balance scores, reaction time, omission, and commission numbers. There were no statistically significant differences in dynamic and static balance scores and in CPT parameters between conditions. In conclusion, there was no immediate neuromodulation effect of anodal ctDCS to improve balance performance in healthy, young individuals. Furthermore, no evidence was found to support the use of cerebellar tDCS to improve sports performance.

## 1. Introduction

Postural control, motor adaptation, and balance are important motor features that are being investigated in terms of rehabilitation and performance enhancement [1,2,3,4]. Maintaining the static and dynamic balance of the body is a crucial function of the nervous system for both movements in our daily life, such as standing and walking, and adaptation to rapid changes in body posture. Therefore, improvements in the balance of patients with a high risk of falls is a crucial clinical goal; nowadays, neuromodulation techniques are increasingly being investigated for possible boosting effects [5,6,7]. Additionally, taking into consideration the research on healthy individuals and the importance of balance in sports, we can conclude that improving balance performance may have a potential for performance enhancement in several sports in the future [8,9]. Based on the value of those functions, transcranial direct current stimulation (tDCS) may be a potential tool for an improvement in balance and motor neurorehabilitation in balance disorders. The modulation of balance with tDCS has shown positive results in patients with neurological diseases—such as stroke, Parkinson’s disease, and ataxia—and older adults who have a high risk of falls [7,8,10,11,12,13,14,15,16]. The commonly used target areas for tDCS with the aim of balance improvement are the motor cortex and cerebellum [17].

Balance-related tDCS studies targeting the cerebellum still seem to be heterogeneous. If only research conducted on healthy volunteers is considered in relation to our study, contradictory results in terms of effect and polarity are found [18,19,20,21,22]. While some studies have found significant impairment in balance with cathodal cerebellar tDCS [19], some have found improvement [21,23]. Two other studies have reported an improvement in balance with anodal cerebellar tDCS [18,24]. Also, there are studies that have reported no significant effect of cerebellar tDCS on balance or minimal measure-specific effects [20,25,26]. The bias toward publishing positive results and the lack of reproducibility together with contradictory findings directed us to design a study to replicate and investigate the anodal cerebellar tDCS effect on balance in healthy, young adults. Furthermore, any side effect on cognitive and motor functions could be a drawback to the usage of tDCS with an aim for balance improvement. In consideration of the tDCS literature, our primary aim was to investigate the effects of cerebellar anodal tDCS on dynamic and static balance in healthy, young adults. We designed a single-session anodal cerebellar tDCS study on healthy, young adults in comparison with sham stimulation and evaluated its effect on dynamic and static balance tests, which are the most common measurements in sports for balance measurement, the Y-Balance and Flamingo tests [27]. Most tDCS studies have used a stability platform for balance measurements, and a few of them included static balance measurements. According to our knowledge, only one recent study used a Y-Balance test to detect the modulation effect of single-session tDCS and found a positive effect of motor cortex stimulation in healthy, young adults [28]. Therefore, in this study, we were able to see the effects of cerebellar tDCS on the Y-Balance and Flamingo tests in healthy subjects for the first time. These tests are commonly used for balance performance measurement and balance training in young athletes. If we are able to detect a modulation effect by using them, instead of non-portable and costly balance measurement methods that require specialization for data analysis, we can utilize simple, inexpensive, and portable tests in future neuromodulation investigations on healthy, young adults/athletes. The changes in impulsivity, attention, and reaction time (RT) were also measured using a Continuous Performance Test (CPT). CPT is a task-based and computerized test for the evaluation of attention, impulsivity, and vigilance. It was originally used by Rosvold in 1956 [29] for the measurement of sustained attention, and various versions of CPT were developed in the following years. It is now the most common test for the measurement of attention in clinics and research [30]. We aimed to not miss any adverse effect on cognition and reaction functions via the use of tDCS with the aim of balance performance enhancement.

## 2. Materials and Methods

### 2.1. Subjects

Twenty-one healthy, young individuals aged between 18 and 22 (mean age, 20.12 ± 1.05 years; 10 males) volunteered to participate in the study. None of them had a history of neurological, psychiatric, or orthopedic diseases/disorders or used any medication affecting the nervous system. Volunteers who exercised regularly (especially in sports that require balance training, such as gymnastics, yoga, board sports, etc.), who were professional athletes, or who had any metal implants or hip/knee prostheses were excluded from the study. Three participants’ data were excluded from the analysis due to their high scores on the Beck Depression Inventory (BDI) (above 19) and the Beck Anxiety Inventory (BAI) (above 15), which indicate moderate–severe depression and anxiety. The validated Turkish versions of these inventories were used [31,32]. They were also non-smokers and had not consumed alcohol for at least 24 h before testing. Each participant read and signed written informed consent, and they were given detailed information about the study. The study was conducted according to the Helsinki Declaration 2013 version and approved by the Istinye University Ethical Committee.

### 2.2. Experimental Design

**Figure 1:**
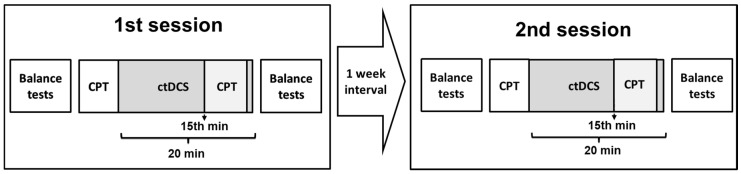
Experimental design. Two ctDCS sessions (anodal ctDCS–sham ctDCS) were applied in a randomized order with a 1-week interval. Balance tests included the Flamingo and Y-Balance tests. CPT; Continuous Performance Test.

The experimental design is shown in Figure 1. After receiving informed consent, participants were asked to complete the Waterloo Foot Dominance Test (FDT), BDI, and BAI inventories [33]. Before the balance tests, some basic stretching exercises were performed to maintain warm-up and prevent the muscles from being damaged. Respectively, the Flamingo test (Eurofit test battery) and Y-test were performed for balance measurements. After the balance tests, participants were asked to do the CPT. The subjects performed the CPT for 30 s for familiarization, followed by a complete CPT lasting approximately 4.5 min. The tDCS application began immediately after participants completed the CPT. The participants were blinded to the type of tDCS (sham or active). Participants repeated the CPT, starting at the 15th minute of tDCS application (total time 20 min). After tDCS was completed, the balance tests were repeated. Subjects were recruited to the same experimental stages in the second session after one week. All participants received both types of stimulation (anodal and sham) in a randomized order in the same experimental steps, with a one-week interval. The randomization was conducted using the web-based algorithm “www.randomization.com (accessed on 15 January 2019)”.

### 2.3. Cerebellar tDCS

**Figure 2:**
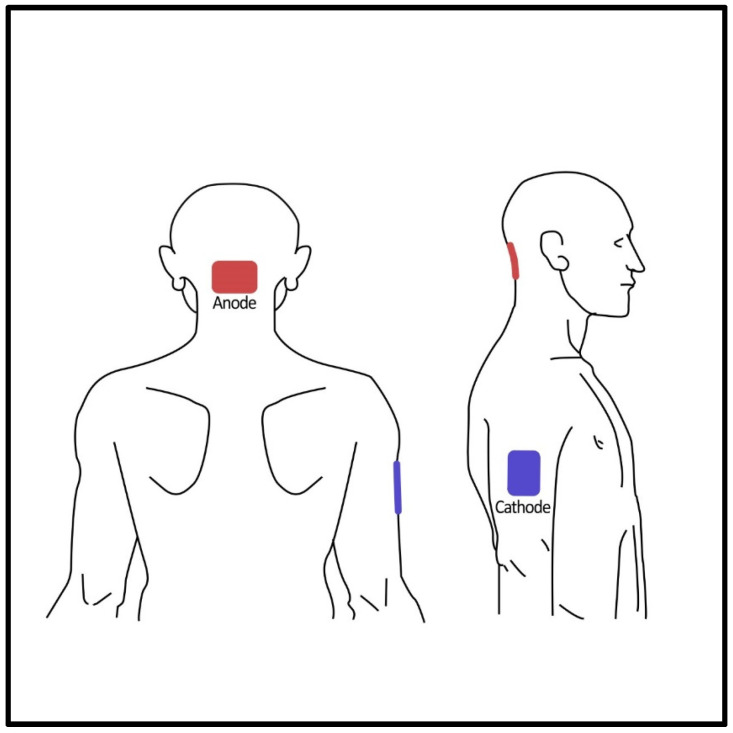
Posterior and lateral views of the electrode positioning.

Cerebellar tDCS was delivered using a direct current stimulator (DC-Stimulator, NeuroConn GmbH, Ilmenau, Germany) via two saline-soaked rectangular sponge electrodes (5 × 7 cm). The anode electrode was placed over the cerebellar vermis (2 cm below the inion) while the reference electrode was placed over the right upper arm (Figure 2). The most important reason for preferring the shoulder montage is to eliminate the possible influence of the cathode electrode. Because even though the standard reference electrode locations are more or less clear in bicephalic montages, the possible influence of the reference electrode is always a challenging factor to consider. Parazzini and colleagues conducted a modeling study about cerebellar tDCS and showed that this electrode montage was efficient in stimulating the cerebellum while no spread was found in the brainstem and heart [34]. The tDCS was applied for 20 min at a current intensity of 2 mA with 30-s fade-in and fade-out periods. The sham procedure was 1 min of active stimulation at the beginning of the session, with the same electrode montage and fade-in and fade-out periods. It was repeated in the 10th minute of the session. At the end of the tDCS sessions, participants were asked to guess whether they received an active or sham stimulation. Any sensations such as itching, burning, or discomfort under the electrodes during tDCS were recorded.

### 2.4. Continuous Performance Test (CPT)

The basic CPT procedure consists of a rapid presentation of consistently changing visual stimuli that includes a target to which the subject must respond. In our study, the total number of stimuli was 300, and the CPT lasted approximately 4.5 min. The entire CPT process was performed in front of the screen in a silent lab environment. The subjects were asked to press the spacebar only when they saw the letter “A” (target) following the letter “Z”. The number of responses to the stimuli other than the target stimulus was counted as commissions as a measure of impulsivity. The times that the participant remained unresponsive to the target stimulus were counted as omissions as a measure of attention. The number of omissions (missed targets), commissions (responses to false stimuli), and reaction times (correct response latency) in milliseconds were calculated.

### 2.5. Balance Tests

The subjects performed two separate balance tests: the Y-Balance test to assess dynamic balance and the Flamingo Test to assess static balance [35,36,37]. Verbal instructions were given to participants on how to perform the tests before they began. Thereafter, the subjects completed 2 min of warm-up movements that were chosen beforehand based on the nature of the assessments. After the warm-up, the subjects were asked to practice test trials to familiarize themselves with the procedure before taking actual measurements. Following the practice trials, the subjects had a 2 min rest period before the measurements. The balance tests were performed before and after each tDCS session in the following order: warm-up, Flamingo test, and then Y-Balance test. The Flamingo test was performed before the Y-Balance test to avoid interfering with the results of the latter, as the Flamingo test generates less muscle fatigue. 

**Figure 3:**
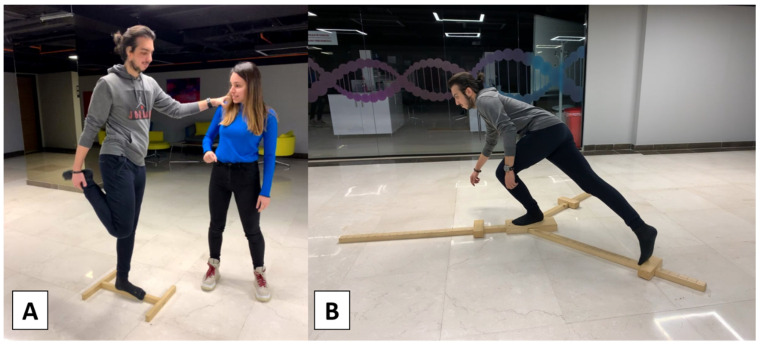
Static and dynamic balance tests; (**A**): Flamingo test, (**B**): Y-Balance test.

The Flamingo Balance Test was used to assess the ability to balance successfully on a single leg and assess the participants’ static balances. A 50 cm long, 5 cm high, 3 cm wide beam (stabilized with two supports at each end and with a non-slip surface) and a timer were required for the test. Participants were instructed to take off their shoes and stand on one foot on the balance beam, with the free leg flexed at the knee and the foot held close to the buttocks (with the help of the same-sided hand while the other one was free for balancing—Figure 3). In the beginning, the participants were allowed to maintain their balance by holding the instructor’s hand. As soon as the participant let go of the instructor’s hand, the stopwatch was started. The stopwatch was paused every time the participant fell off the beam or let go of their flexed leg and resumed when they returned to the testing position. This procedure continued until the participant’s total standing time reached 60 s. The instructor took notes on the testing table each time the stopwatch was stopped. The participant’s score was determined by the number of falls in 60 s. The test was repeated for both legs. Lower Flamingo balance scores indicated a better whole-body static balance.

After the Flamingo test, the Y-Balance test was conducted. The Y-Balance test was developed based on the Star Excursion Balance Test (SEBT). The SEBT assesses the ability to maintain balance on one leg while the contralateral leg reaches as far as possible in eight directions. In the Y-Balance test, the directions are reduced to three, which shortens the evaluation time. The Y-Balance test requires the participant to balance on one leg whilst pushing a small indicator block as far as possible in three separate directions: anterior, posterolateral, and posteromedial with the other leg (Figure 3). To reduce the learning effect, the participants were first instructed to try all three directions six times with each leg. The participants were not allowed to lose contact with the block, touch the ground with their foot, use the block for stance support, or touch the ground with their reaching foot before returning to the starting position under control. Otherwise, the trial was not considered successful during the test. Following the trial, the participants started with a right-leg stance and pushed the block as far as possible in the anterior direction. If the movement was successful, the instructor recorded the length between the block and the platform. After three successful trials, the participants were asked to do the same with the left leg. When the participants completed the anterior direction, they were asked to continue with the right leg in the posteromedial direction and the next posterolateral direction. Three successful attempts for each direction and both legs were recorded for the calculation. The reach distances were normalized to the limb length, measured from the anterior superior iliac crest to the medial malleolus. The composite score used to determine the results was the sum of the maximum reach distances in the three directions, divided by three times the lower limb length, and multiplied by 100. The composite score was calculated for both legs.

## 3. Results

Twenty-one participants were recruited for the study; however, three participants were excluded due to high BDI/BAI scores. Therefore, the data from 18 subjects were included in the statistical analysis. The repeated measures ANOVA were used to compare the differences in changes in the balance test scores (dominant and non-dominant leg), reaction times, omissions, and commissions between active and sham tDCS conditions. The differences were considered to be significant at *p* < 0.05. There was no statistically significant difference in the Y-Balance test score changes between anodal and sham stimulation for the dominant foot (F(1,17) = 0.152, *p* = 0.702) and also for the non-dominant foot (F(1,17) = 0.075, *p* = 0.787). There was no statistically significant difference in the Flamingo test score changes between anodal and sham stimulation for the dominant foot (F(1,17) = 0.209, *p* = 0.654) and also for the non-dominant foot (F(1,17) = 0.036, *p* = 0.851). The changes of commissions (F(1,17) = 0.209, *p* = 0.654) and omissions (F(1,17) = 0.036, *p* = 0.851) in the CPT did not differ between stimulation groups. On the other hand, there was a tendency to delay in mean reaction times in anodal stimulation compared to sham stimulation, but it did not reach statistical significance (F(1,17) = 2.544, *p* = 0.129) (Table 1). Only 5 of the 18 participants correctly identified the type of stimulation they received, which indicates a good sham procedure. No serious adverse effects occurred. Two participants reported a mild headache, and one participant reported an itching sensation under the electrodes.

## 4. Discussion

In the present study, we investigate the effect of single-session anodal cerebellar tDCS on dynamic and static balance in healthy, young adults, together with the changes in impulsivity, attention, and reaction times. The results did not suggest any significant change in both balance tests immediately after anodal cerebellar tDCS, although there was a tendency to increase in reaction times without any change in total omission and commission numbers. Furthermore, at the end of the study, only five participants were able to correctly guess whether they were receiving an active tDCS or sham tDCS, supporting that our sham stimulation technique was successful in blinding the subjects. One possible reason for failure in modulation could be the method of tDCS in our study, which might not be efficient in affecting the cerebellum (parameters, montage, number of sessions, target area, etc.). Therefore, we will discuss the methodological differences by comparing them with the literature.

When single-session studies with favorable results in healthy subjects were examined, only a few research came forward [15,18,21]. In a study by Ehsani and colleagues, the participants were older adults. They applied 20 min of 1.5 mA anodal tDCS over the cerebellum and evaluated static and dynamic balance before and immediately after tDCS. The electrode montage was the same as in our study with a slight difference in the sizes of electrodes (they used 5 × 5 cm). The results revealed an improvement in both static and dynamic balance and postural stability indices immediately after anodal cerebellar tDCS. They concluded that anodal cerebellar tDCS would improve balance in older adults. This conclusion was coherent since the correlation between cerebellar vermis volume reduction and age-related balance deficits has been shown in several studies [38,39,40]. The researchers emphasized that the successful balance modulation in their study might be due to the facilitated cerebellar connectivity via improvement in vermis functions, and it might be able to compensate for age-related changes and modulate the balance performance in older adults. The difference in tDCS methodology was only the lower intensity of current (1.5 mA). Therefore, in comparison with Ehsani’s study, we may conclude that the same tDCS protocol is not efficient for healthy, young subjects. Additionally, the explanation above, on the other hand, leads to the conclusion that anodal cerebellar tDCS may work in pathological conditions involving the cerebellum; however, it is obvious that cerebellar neuromodulation with tDCS does not work in such a straightforward inference. Although there are many studies on the positive effect of anodal cerebellar tDCS on visuomotor adaptation and motor learning, Jalali et al. failed to validate their initial positive effect in a second set of participants and questioned the consistency of the effect of cerebellar tDCS on functional tasks and the validity of using it within a clinical context [41]. Furthermore, there are also studies in the literature that failed to show a positive modulatory result neither in an acute nor in a long-term application of anodal cerebellar tDCS in Parkinson’s disease, in which the cerebellum is involved in its pathophysiology [42,43].

Another study that resulted in a successful balance modulation in healthy subjects with single-session cerebellar tDCS is Poortvliet and colleagues’ research in 2018 [24]. In that study, anodal cerebellar tDCS reduced forward displacement and variability in the center of pressure (COP) against Achilles tendon vibration in healthy adults. The current intensity was 1 mA, and a large (10 × 10 cm) cathode electrode was placed on the forehead, which were the main differences from the current study. Furthermore, they used a sensory perturbation for postural balance measurements. These differences in tDCS applications and study designs cause difficulty to compare and interpret the results.

In a recent study in 2020, the effect of tDCS, visual feedback, and their combination on balance control was investigated in healthy participants [21]. The results conflicted with the aforementioned studies. While an improvement was found in balance with cathodal cerebellar tDCS, no statistically significant change was observed in the anodal tDCS group, both during and after the session. The improvement in balance with cathodal tDCS was an online effect (during tDCS), which was not significant after the intervention. It is not possible to compare our results with theirs since we did not measure during the tDCS sessions.

While heterogeneous methodologies create difficulty in comparing the results of the studies, the conflicting results prevent a clear conclusion about the modulatory effect of cerebellar tDCS. Several studies did not show cerebellar modulation effect in healthy subjects [19,20,25,44]. A research group presented their findings which showed no effect of cerebellar tDCS on complex whole-body dynamic balance tasks in both young and older healthy subjects, even after anodal and cathodal stimulation [20,25,44]. In their latest study, they changed the orientation of the electrode and again did not find an effect. Two studies with healthy participants found conflicting results. Foerster and colleagues showed impairment in postural stability scores after cathodal stimulation; on the other hand, Inukai and colleagues showed decreased body sway after cathodal cerebellar tDCS [19,23]. Those conflicts make it complicated to draw a precise conclusion.

A recent systematic review analyzed the studies that investigated the effects of tDCS on balance according to different balance measurements—static and dynamic [45]. They reported that there was a lack of evidence on the effect of cerebellar tDCS on static balance in young people; on the other hand, there was a positive effect on both static and dynamic balance in healthy older adults. The conclusion about the effect on healthy older adults was supported by a study published recently [46]. It has been shown that there are changes in the integrity and volume of the cerebellum and vermis which may be the cause of age-related balance problems. Concerning the literature, the authors suggested that anodal cerebellar tDCS may enhance the function of Purkinje fibers and vermis and improve the activity of cerebellar connections, which is in line with Ehsani’s conclusion mentioned above. Therefore, it can be suggested for use in older adults for balance improvement. However, there was no clear conclusion about its effect on healthy, young individuals due to a lack of studies and evidence, and our results support that “aging” is a possible important determining factor for the modulatory effect of cerebellar tDCS. Although highly trained athletes were excluded from the study design, the lack of a modulatory effect of tDCS in healthy, young subjects may be due to the “ceiling effect”. Trying to improve an already good performance may indicate that the robust physiological mechanism is at its maximum, making it impossible to further enhance the performance of the mechanism.

One can question the balance measurement methods of our study and whether they were sensitive enough to detect the changes and were challenging enough to detect the differences. In some of the aforementioned studies, the Biodex Balance System was used to measure postural stability, and both stability indices and the Berg Balance Score changed after anodal tDCS. Since our subjects were healthy, young individuals, we chose relatively challenging methods (Flamingo and Y-Balance), which have been shown to be reliable for the measurement of balance in young athletes [36]. They are especially used in sports to track the improvement of athletes who have better balance abilities than older adults.

The tendency towards prolongation of reaction time in the current study is compatible with the literature. Ferrucci and colleagues found an impairment in practice-dependent improvement in reaction times after both cathodal and anodal cerebellar tDCS [47]. On the other hand, in a more recent study, three types of cerebellar tDCS conditions (anodal, cathodal, and sham) were compared with a serial reaction time task, and an increase in reaction times was found in anodal ctDCS [48]. They concluded this as a strengthening of the cerebellum’s inhibitory effect on motor pathways with anodal stimulation. Since we did not have cathodal stimulation, we could not investigate the polarity-specific effect on reaction times; however, our result even though not statistically significant, supports a prolongation in anodal stimulation compared to sham.

There are two limitations in the current study. First, a single-session tDCS was used, which cannot represent the effects of a multi-session application. Therefore, multi-session studies are needed to investigate long-term effects. The second limitation is the timing of the measurement. We measured the static and dynamic balance immediately after the tDCS sessions, so it was not possible to interpret the online effect of it and compare the results with the studies that investigated the online effect. If we criticize our study, we may say that adding postural control measurements on the balance platforms, besides the Flamingo and Y-Balance tests, would increase the power and reliability of the results.

## 5. Conclusions

The results of the studies investigating the effect of cerebellar tDCS on balance in healthy subjects are conflicting, and the reviews also suggest contradictory conclusions. The positive effect of tDCS on postural control in patients and older adults in previous studies was not observed in healthy, young individuals in the current study. On the other hand, even though the number of errors did not increase, the delay in reaction time is not a desired effect for performance enhancement. As a result of the study, there was no effect of single-session anodal cerebellar tDCS on dynamic and static balance performances of healthy, young individuals. Additionally, if the use of cerebellar tDCS for balance performance enhancement is aimed at healthy subjects, the effect on reaction times must be considered carefully, as it is also an important factor for activity performance.

## Figures and Tables

**Table 1:** Means of composite scores of Y-Balance and Flamingo tests before and after tDCS sessions. Mean omission, commission, and reaction time (RT) of Continuous Performance Test (CPT). D; dominant, ND; non-dominant.

**Anodal tDCS**							
**Tests**	**Y-Balance scores**		**Flamingo scores**		**CPT results**		
**Foot**	D	ND	D	ND	Omission	Commission	RT (ms)
**Pre**	81.12 ± 5.33	81.35 ± 5.37	13.33 ± 3.94	14.44 ± 5.83	0.61 ± 0.91	1.22 ± 1.21	317.94 ± 55.76
**Post**	82.72 ± 6.35	82.37 ± 6.72	11.38 ± 4.40	12.55 ± 4.28	0.27 ± 0.57	0.83 ± 0.92	**332.88 ** **± 50.40**
**Sham tDCS**							
**Tests**	**Y-Balance scores**		**Flamingo scores**		**CPT results**		
**Foot**	D	ND	D	ND	Omission	Commission	RT (ms)
**Pre**	81.63 ± 5.34	82.84 ± 5.17	13.33 ± 5.26	13.33 ± 5.22	0.27 ± 0.46	1.16 ± 1.38	316.72 ± 48.60
**Post**	82.05 ± 5.97	83.30 ± 6.32	10.88 ± 4.30	11.16 ± 5.46	0.22 ± 0.42	0.88 ± 0.83	**321.83 ** **± 50.17**

## Data Availability

The data presented in this study are available on request from the corresponding author.
